# Respiratory DNA viruses are undetectable in nasopharyngeal secretions from adenotonsillectomized children

**DOI:** 10.1371/journal.pone.0174188

**Published:** 2017-03-17

**Authors:** Ronaldo Bragança Martins, Lucas Penna Rocha, Mirela Moreira Prates, Talita Bianca Gagliardi, Balduino Biasoli, Marcelo Junqueira Leite, Guilherme Buzatto, Miguel Angelo Hyppolito, Davi Casale Aragon, Edwin Tamashiro, Fabiana Cardoso Pereira Valera, Eurico Arruda, Wilma Terezinha Anselmo-Lima

**Affiliations:** 1 Departments of Cell Biology, School of Medicine of Ribeirão Preto of University of São Paulo, Ribeirão Preto, Brazil; 2 Ophthalmology, Otorhinolaryngology and Head and Neck Surgery, School of Medicine of Ribeirão Preto of University of São Paulo, Ribeirão Preto, Brazil; 3 Department of Pediatrics, School of Medicine of Ribeirão Preto of University of São Paulo, Ribeirão Preto, Brazil; Kliniken der Stadt Köln gGmbH, GERMANY

## Abstract

Respiratory viruses are frequently detected in association with chronic tonsillar hypertrophy in the absence of symptoms of acute respiratory infection (ARI). The present analysis was done in follow-up to a previous clinical study done by this same group. Nasopharyngeal washes (NPWs) were obtained from 83 of 120 individuals at variable times post adenotonsillectomy, in the absence of ARI symptoms. A look back at virus detection results in NPWs from the same 83 individuals at the time of tonsillectomy revealed that 73.5% (61/83) were positive for one or more viruses. The overall frequency of respiratory virus detection in post-tonsillectomy NPWs was 58.8%. Rhinovirus (RV) was the agent most frequently detected, in 38 of 83 subjects (45.8%), followed by enterovirus in 7 (8.4%), human metapneumovirus in 6 (7.2%), human respiratory syncytial virus in 3 (3.6%) and human coronavirus in 1 (1.2%). Remarkably, there was no detection of adenovirus (HAdV) or human bocavirus (HBoV) in asymptomatic individuals in follow-up of adenotonsillectomy. In keeping with persistence of respiratory DNA viruses in human tonsils, tonsillectomy significantly reduces asymptomatic shedding of HAdV and HBoV in NPWs.

## Introduction

A wide range of respiratory viruses can be detected in human adenoids and palatine tonsils [1, 2, 3, 4]. A previous cross-sectional study of hypertrophic tonsils and nasopharyngeal secretions from patients undergoing adenotonsillectomy revealed very high frequencies of detection of respiratory viruses by PCR [1]. Overall, 97% of 120 individuals without symptoms of acute respiratory infection (ARI) had at least one respiratory virus detected by RT-PCR in either adenoid, palatine tonsil, or nasopharyngeal washes (NPWs). Considering only NPWs, 78.5% were positive for one or more viruses [1]. Those results prompted us to assess whether asymptomatic shedding of respiratory viruses is also frequent in asymptomatic individuals whose tonsils were removed. To address that question, NPWs collected from asymptomatic subjects in follow-up of adenotonsillectomy [1] were tested for a comprehensive respiratory virus panel.

## Materials and methods

The study was conducted according to the principles expressed in the Declaration of Helsinki and was approved by the Ethics Review Committee of the Hospital das Clínicas de Ribeirão Preto (Number 10466/2008) and a written informed consent was obtained from all parents and guardians prior to enrollment.

Parents and guardians of 120 patients enrolled in the previous study [1] were actively contacted by phone 3 to 5 years (mean 4.2 years) after undergoing surgical removal of both adenoid and palatine tonsils at the Division of Otorhinolaryngology of the Clinical Hospital of the University of São Paulo School of Medicine, in Ribeirão Preto, Brazil. Eighty-three of 120 patients (46% males) with ages ranging from 6 to 17 (mean 11, median 10) years, accepted to participate in this study. All participants had previously undergone adenotonsillectomy, and were seen in follow-up visits at the Otolaryngology outpatient clinic and, after an informed consent was obtained from parents/guardians, NPWs were performed by a physician, as previously described [1].

NPWs from a control group consisting of 20 asymptomatic children (65% males) were tested. Those children, with ages ranging from 1 to 12 (mean 4.2, median 3) years, underwent surgery for cochlear implant. All children in the control group had normal size tonsils at the time of sampling. For all patients NPWs were done with saline solution, immediately placed on ice for transportation to the laboratory, within one hour. Similarly to the previous study, exclusion criteria, for both the study and control groups on the present analysis, were presence within one month prior to visit of symptoms of acute respiratory infection including fever, runny nose, stuffy nose and sore/scratchy throat; or antibiotic treatment.

In the laboratory, NPWs were aliquoted in Trizol (Invitrogen, Carlsbad, CA, USA) and kept at—80°C until further testing. Total nucleic acids were extracted from NPWs following Trizol manufacturer’s instructions. For RNA virus detection, reverse transcription was carried out prior to real-time PCR using 1 μg of extracted RNA primed with random hexamers and Multiscribe reverse transcriptase (Applied Biosystems, Foster City, CA, USA), following the protocol proposed by the manufacturer. All PCR assays were done on a Step One Plus Real-Time PCR equipment (Applied Biosystems, Foster City, CA,USA), with the same set of primers and probes published before [1], except for RV detection, for which primers and probes were designed covering RV species A, B and C (S1 Table). Real-time PCR for the RNAse-P housekeeping gene was done as endogenous control. Adequate positive and negative controls were included in all real-time PCR batches. Associations were tested by Fisher’s exact test, considering a significance level of 5%.

## Results and discussion

A look back to the previous results of virus detection obtained for the same 83 patients [1] revealed that at least one virus had been detected in 73.5% of the NPWs collected immediately prior to tonsillectomy. At that time, respiratory viruses were detected in 90.3% of adenoids and 77.1% of palatine tonsils from the same 83 patients. The overall frequency of virus detection in the NPWs from the same individuals a few years after adenotonsillectomy was 58.8%, and all patients were free of symptoms of acute respiratory infections for at least one month prior to sample collection. The overall rate of viral detection in samples from 20 control individuals was 14/20 (70%).

Considering only the 83 patients who accepted to participate in the follow-up analysis, the most frequent virus in preoperative NPWs was RV, detected in 27 (32.5%) subjects, followed by HAdV in 19 (22.9%), EV in 18 (21.7%), HRSV in 9 (10.8%), and HMPV, HBoV, HCoV and FLU, detected in frequencies lower than 10% each. Except for RV, the frequencies of detection of each specific virus were lower in follow-up samples than in those obtained at the time of surgery (Table 1). The follow-up detection rates were 45.8% (38/83) for RV, 8.4% (7/83) for EV, 7.2% (6/83) for HMPV, 3.6% (3/83) for HRSV and 1.2% (1/83) for HCoV; whereas HAdV and HBoV were not detected in any of the 83 subjects. Considering DNA viruses in the control group, HAdV was detected in 5 of 20 (25%) NPWs, and HBoV in 2/20 (10%). Frequencies of RNA viruses were 15% for EV, 5% for RV, 15% for RSV, 5% for HMPV and 5% for Flu-B virus (S2 Table). The similar frequencies of detection of respiratory viruses between NPWs from control group subjects and those obtained pre-operatively from patients with adenotonsillar hypertrophy, indicate that respiratory viruses seem not to be associated with pathogenesis of hypertrophic tonsillar disease. Rather, these similarly high frequencies of genome detection indicate that adenoid and palatine tonsils, hypertrophic or not, may serve as reservoirs of respiratory viruses that are shed in NPWs.

**Table 1:** Respiratory viruses detected by real-time PCR in NPWs from 83 patients pre- and post-adenotonsillectomy.

Respiratory Viruses	NPWs collected preoperatively*	NPWs collected post-tonsillectomy	p-value**
RV	27 (32.5%)	38 (45.8%)	0.12
HAdV	19 (22.9%)	0 (0%)	< 0.01
EV	18 (21.7%)	6 (7.2%)	0.02
HRSV	9 (10.8%)	1 (1.2%)	0.13
HMPV	8 (9.6%)	3 (3.6%)	0.78
HBoV	6 (7.2%)	0 (0%)	0.02
HCoV	3 (3.6%)	1 (1.2%)	0.62
FLU A	1 (1.2%)	0 (0%)	1
FLU B	0 (0%)	0 (0%)	1
HPIV-I	0 (0%)	0 (0%)	1
HPIV-III	0 (0%)	0 (0%)	1

* N (%) of positive samples.

** Fisher’s exact test.

The frequencies of RV detection pre- and post-adenotonsillectomy were not significantly different. Of note, 31.6% (12 of 38) of the patients positive for RV in the follow-up study had also been positive in the preoperative study. The frequencies of patients repeatedly positive for viruses of the same species were 57% (4 of 7) for EV and 50% (3 of 6) for HMPV. RV was co-detected with other viruses in 5 cases (5.8%), 3 with HMPV and 2 with EV, indicating a significant reduction in virus co-detections rates as compared to those obtained preoperatively for the same individuals (45.8%).

Remarkably, none of the samples collected in follow-up of adenotonsillectomy were positive for HAdV or HBoV. The lack of detection of HAdV in follow-up NPWs represented a significant reduction from the rate of 22.9% in the preoperative analysis (p-value< 0.01). However, it should be kept in mind that results of lower virus detection rates post-adenotonsillectomy could also have been affected by an overall state of better health and reduced rates of respiratory infections in general in children after tonsillectomy. In addition, in the follow-up analysis children have become older, and this may have affected age-related susceptibilities to such viruses as HAdV and HBoV, which are generally more common in smaller children and toddlers [5, 6, 7].

Detection of respiratory viruses in tonsillar tissue is significant, not only because it may complicate laboratory diagnosis of viral ARI, but especially because those tissues can be potential sources of infectious viruses in the community. By analogy, the role of tonsils, and especially of tonsillar B lymphocytes as reservoirs of Epstein-Barr virus is well established [8]. Viruses produced in tonsillar tissues and excreted into respiratory secretions can disseminate not only to other tissue (e.g., lower respiratory tract, middle ear and paranasal sinuses), but also to other subjects [9, 10, 11].

Adenovirus received its name because it was initially recovered from adenoid [12], where it can establish long-lasting infection [13, 14]. This is especially true of adenovirus of species C (types 1, 2, and 5), followed by types 3 and 7 of species B, HAdV-E4 and HAdV-F4 [15]. Therefore, detection of HAdV DNA by PCR in tonsillar tissue is not too surprising. In fact, group C HAdV DNA has been detected in tonsillar lymphocytes in the absence of infectious particles [14]. A look back to the previous results obtained only for the 83 patients enrolled in the present analysis, shows that HAdV had been detected in 22.9% of the NPWs at the time of tonsillectomy [1]. Therefore, the lack of detection of adenovirus in the follow-up analysis is very significant, coming in strong support of the pivotal role of tonsils in HAdV persistence and as potential sources of asymptomatic HAdV shedding in respiratory secretions. Moreover, these findings suggest that detection of HAdV by PCR in respiratory samples may be clinically irrelevant for the diagnosis of ARI in children who have not undergone tonsillectomy.

HBoV is in the family *Parvoviridae*, which includes agents capable of causing persistence, like parvovirus B19, which can persist in both immunocompromised [16] and immunocompetent individuals [3]. A previous study by this group has shown that hypertrophic adenoids commonly harbor HBoV, albeit only 20% of them were actively replicating [17]. That was suggestive that HBoV genomes detected in tonsils were most likely latent as episomal genomes, or as viral DNA concatemers [18, 19]. Consistent with that, HBoV DNA was not detected in any of the 83 individuals after adenotonsillectomy.

Except for RV, RNA virus detection rates were generally lower in samples collected from the same individuals after tonsillectomy, but reductions were generally not significant. Of note, although EV detection rate was significantly reduced (p = 0.02), it was still at 7.2% after tonsillectomy. Therefore, differently from DNA viruses, respiratory RNA viruses were detected in asymptomatic individuals in follow-up of adenotonsillectomy in the present analysis, suggesting thatthey may be long lasting or persisting in tissues other than adenoids and palatine tonsils, and thus continue being shed in respiratory secretions independently of previous adenotonsillectomy. None of the 83 patients studied had recurrence of adenoidal tissue, indicating that the bulk of the adenoid had indeed been removed, but it should be kept in mind that complete adenoidectomy is anatomically impossible, and therefore some remaining lymphoid tissue may have persisted in the nasopharyngeal wall. Therefore, possible sites of infection in these asymptomatic individuals post-adenotonsillectomy could be remnants of adenoids, lingual and tubal tonsils, as well as mucosal-associated lymphoid tissue (MALT).

It has been widely known that RV is the most frequently detected respiratory virus, both in symptomatic and asymptomatic subjects [20, 21]. In asymptomatic subjects, frequencies of RV detection by PCR have varied from 14% to 53% [22, 23]. A study done in Brazil on a restricted number of patients reported similar results, indicating that, after adenotonsillectomy, detection of respiratory viruses in NPWs tends to decrease and RV was the most frequently detected virus, with prevalence in pre- and post-surgery of 33% and 14%, respectively [4]. In the present study, RV detection frequency in pre- and post-adenotonsillectomy were not significantly different, but the post-adenotonsillectomy frequency was extremely high (45.8%). This apparent discrepancy between our data and those by Primo et al, [4] could be partially explained by differences in sensitivities of the q-PCR multiplex protocol used by them [4], as compared to the approach of individual real time PCR assays for each virus used in the present study. Importantly, the real-time PCR assay for RV used in the present study was designed to detect all three species of RV, while the multiplex assay used in the other study [4], does not cover RV os species C. Despite the similar prevalences of DNA-viruses in pre- and post-operative secretions observed by Primo et al. [4], more careful analysis of their results reveals that HAdV and HBoV genomes were not detected in postoperative secretion from patients whose adenoid tissue was positive for these viruses, which is congruent with our results.

Whether the detection of RV in asymptomatic adenotonsilectomized individuals comes from delayed clearance of a previous viral infection, or reflects RV shedding from other inflamed sites where ICAM-I is over expressed, such as MALT, nasal polyps, and airway epithelial cells including paranasal sinuses and nasal ephitelium, remain to be determined [24, 25, 26]. The subjects enrolled in this follow-up study underwent sample collection only once, at variable times post-adenotonsillectomy, and results could have been different if asymptomatic viral excretion was assessed by multiple sampling over time in a long-term follow-up. Despite this caveat, the complete lack of HAdV DNA detection by PCR in a sizeable number of adenotonsillectomized individuals, along with positivity for HAdV in 25% of control children with normal tonsils, indicate that tonsils are important sources of asymptomatic shedding and underscores the importance of tonsillar tissues for the circulation of this important agent.

## Conclusion

Asymptomatic respiratory shedding of HAdV and HBoV is significantly reduced by tonsillectomy, which agrees with persistence of respiratory DNA viruses in human tonsils.

## Supporting information

S1 Table Primers and probes used for qPCR. (PDF)

S2 Table Respiratory viruses detected by real-time PCR in NPWs from 20 patients undergoing cochlear implant. (PDF)
