# Supplementary material for: Respiratory DNA viruses are undetectable in nasopharyngeal secretions from adenotonsillectomized children
Source: PLoS One. 2017 Mar 17;12(3):e0174188. doi: 10.1371/journal.pone.0174188 (PMC5357011; doi:10.1371/journal.pone.0174188)
Supplement: S1 Table — (PDF) [file pone.0174188.s001.pdf]

Table S1. Primers and probes used for qPCR.

Virus or housekeeping gene	Primer	Sequence	Target	Reference
**HRSV**	A21-F	GCTCTTAGCAAAGTCAAGTTGAATGA	N	[1]
	A102-R	TGCTCCGTTGGATGGTGTATT	N	
	APB48	Fam-ACACTCAACAAAGATCAACTTCTGTCATCCAGC-Tamra	N	
	B17-F	GATGGCTCTTAGCAAAGTCAAGTTAA	N	
	B120-R	TGTCAATATTATCTCCTGTACTACGTTGAA	N	
	BPB45	Joe-TGATACATTAAATAAGGATCAGCTGCTGTCATCCA-Tamra	N	
**HMPV**	HMPV-F	GTGATGCACTCAAGAGATACCC	N	[2]
	HMPV-R	CATTGTTTGACCGGCCCCATAA	N	
	HMPV-probe	Fam-CTTTGCCATACTTCAATGAACAAC-Tamra	N	
**HEV**	HEV-F	GCGGAACCGACTACTTTGGG	5´UTR	[10]
	HEV-R	CTCAATTGTCACCATAAGCAGCC	5´UTR	
	HEV-probe	Fam-TCCGTGTTTCCTTTTATTCTTATA-MGB	5´UTR	
**RV**	RV-F	ACMGTGTYCTAGCCTGCGTGG C	5´UTR	Developed in the present study.
	RV-R	GAAACACGGACACCCAAAGTAGT	5´UTR	
	RV-probe	Fam-TCCTCCGGCCCCTGAAT-BHQ1	5´UTR	
**HCoV**	HCoV-F3	TGGCGGGTGGGATAATATGT	Pol	[4]
	HCoV-ocF	CCTTATTAAAGATGTTGACAATCCTGTAC	Pol	
	HCoV-R3	GAGGGCATAGCTCTATCACACTTAGG	Pol	
	HCoV-ocR	AATACGTAGTAGGTTTGGCATAGCAC	Pol	
	HCoV-P2	Fam-ATAGTCCCATCCCATCAA-Tamra	Pol	
	HCoV-Poc	Fam-CACACTTAGGATAGTCCCA-Tamra	Pol	
**HPIV**	Para1F	CATTATCAATTGGTGATGG	HN	[5]
	Para1R	CTTAAATTCAGATATGTATCCTG	HN	
	Para1-probe	Fam-CTTAATCACTCAAGGATGTGCAGATATA-Tamra	HN	
	Para3F	CTCGAGGTTGTCAGGATATAG	HN	
	Para3R	CTTGAGGTTGTCAGGATATT	HN	
	Para3-probe	Fam-AATAACTGTAAACTCAGACTTGGTACCTGACTT-Tamra	HN	
**FLU**	InfA for	GACCRATCCTGTCACCTCTGAC	M	[6]
	InfArev	AGGGCATTYTGGACAAAKCGTCTA	M	
	InfAprobe	Fam-TGCAGTCCTCGCTCACTGGGCACG-BHQ1	M	
	INFB-1	AAATACGGTGGATTAAATAAAAGCAA	HA	[7]
	INFB-2	CCAGCAATAGCTCCGAAGAAA	HA	
	INFB-probe	Vic-CACCCATATTGGGCAATTTCCTATGGC-Tamra	HA	
**HAdV**	HAdV-F	GCCACGGTGGGGTTTCTAAACTT	Hexon	[8]
	HAdV-R	GCCCCAGTGGTCTTACATGCACAT	Hexon	
	HAdV-probe	Fam-TGCACCAGACCCGGGCTCAGGTACTCCGA-Tamra	Hexon	
**HBoV**	HBoV-F	GCACAGCCACGTGACGAA	NP1	[9]
	HBoV-probe	Fam-TGAGCTCAGGGAATATGAAAGACAAGCATCG-Tamra	NP1	
	HBoV- R	TGGACTCCCTTTTCTTTTGTAGGA	NP1	
**RNAseP**	RNAseP F	AGATTTGGACCTGCGAGCG	RNAse P	[6]
	RNAseP R	GAGCGGCTGTCTCCACAAGT	RNAse P	
	RNAseP prob	Fam-TTCTGACCTGAAGGCTCTGCGCG-BHQ1	RNAse P	

REFERENCES

1. Hu A, Colella M, Tam JS, Rappaport R, Cheng SM (2003) Simultaneous detection, subgrouping, and quantitation of respiratory syncytial virus A and B by real-time PCR. J Clin Microbiol 41: 149-154.
2. Bouscambert-Duchamp M, Lina B, Trompette A, Moret H, Motte J, et al. (2005) Detection of human metapneumovirus RNA sequences in nasopharyngeal aspirates of young French children with acute bronchiolitis by real-time reverse transcriptase PCR and phylogenetic analysis. J Clin Microbiol 43: 1411-1414.
4. Kuypers J, Martin ET, Heugel J, Wright N, Morrow R, et al. (2007) Clinical disease in children associated with newly described coronavirus subtypes. Pediatrics 119: e70-76.

5. Garbino J, Gerbase MW, Wunderli W, Deffernez C, Thomas Y, et al. (2004) Lower respiratory viral illnesses: improved diagnosis by molecular methods and clinical impact. Am J Respir Crit Care Med 170: 1197-1203.
6. CDC (2009) Protocol of real time RT-PCR for influenza A. Available: http://www.who.int/csr/resources/publications/swineflu/realtimeptpcr/en/index.html. Accessed 22 Februaryt 2012
7. van Elden LJ, Nijhuis M, Schipper P, Schuurman R, van Loon AM (2001) Simultaneous detection of influenza viruses A and B using real-time quantitative PCR. J Clin Microbiol 39: 196-200.
8. Heim A, Ebnet C, Harste G, Pring-Akerblom P (2003) Rapid and quantitative detection of human adenovirus DNA by real-time PCR. J Med Virol 70: 228-239.
9. Neske F, Blessing K, Tollmann F, Schubert J, Rethwilm A, et al. (2007) Real-time PCR for diagnosis of human bocavirus infections and phylogenetic analysis. J Clin Microbiol 45: 2116-2122.
10. Proenca-Modena JL, Pereira Valera FC, Jacob MG, Buzatto GP, Saturno TH, Lopes L, Souza JM, et al. High rates of detection of respiratory viruses in tonsillar tissues from children with chronic adenotonsillar disease. PLoS One. 2012;7(8):e42136.
