# Supplementary material for: Respiratory DNA viruses are undetectable in nasopharyngeal secretions from adenotonsillectomized children
Source: PLoS One. 2017 Mar 17;12(3):e0174188. doi: 10.1371/journal.pone.0174188 (PMC5357011; doi:10.1371/journal.pone.0174188)
Supplement: S2 Table — (PDF) [file pone.0174188.s002.pdf]

Table S2. Respiratory viruses detected by real-time PCR in NPWs from 20 patients undergoing cochlear implant

Respiratory Viruses	NPWs collected preoperatively N(%)
**RV**	1 (5%)
**HAdV**	5 (25%)
**EV**	3 (15%)
**HRSV**	3 (15%)
**HMPV**	1 (5%)
**HBoV**	2 (10%)
**HCoV**	0
**FLU A**	0
**FLU B**	1 (5%)
**HPIV-I**	0
**HPIV-III**	0
